# The Placode Lineage Contributes to the Enteric Nervous System: A Caution for Cell Transplantation Therapy for Hirschsprung Disease

**DOI:** 10.1016/j.jcmgh.2025.101657

**Published:** 2025-10-03

**Authors:** Shigeru Sato, Henry M. Sucov, Takako Makita

**Affiliations:** Division of Biology, Center for Molecular Medicine, Jichi Medical University, Shimotsuke, Tochigi, Japan; Department of Regenerative Medicine and Cell Biology, Medical University of South Carolina, Charleston, South Carolina

The enteric nervous system (ENS) forms during development from progenitors that migrate through the fetal gut from cranial sites of origin. A deficiency in progenitor cell migration and gut colonization results in aganglionosis in the terminal bowel. After birth, when fecal progression through the hindgut requires a functional ENS circuitry, inadequate ENS function leads to inability to defecate and involuntary fecal retention. This condition is called Hirschsprung disease (HSCR). In the clinic, surgical resection of the aganglionic portion of the bowel is the only available treatment, although complications and persisting undesirable bowel function are common.^1^

Because HSCR is a developmental manifestation of incomplete neurogenic colonization, new strategies that involve transplantation of enteric progenitors into the aganglionic segment of the hindgut are under active investigation. For such cell-based therapies to successfully recreate a functional colonic circuitry, it is essential that the developmental biology of the hindgut ENS be properly understood. Cell transplantation approaches that do not incorporate the correct developmental insights may fail or may provide suboptimal outcomes.

The entirety of the ENS has long been thought to arise exclusively from the neural crest, a migratory population of multipotent progenitors that delaminate from the dorsal-most aspect of the neural tube during early development.^2^ We recently provided evidence in mice that the embryonic placodes are a second and independent source of ENS progenitors.^3^ Placodes are similar to neural crest in that both generate migratory neurons and other cell types,^4^ but the placode lineage had never before been associated with the mammalian ENS. In our study,^3^ we used Wnt1Cre to label the neural crest cell lineage, and Pax2Cre to label the placodal lineage. These Cre lines are very well-characterized and support highly efficient recombination that is authentic to the neural crest or placode lineages, as defined by many prior studies. We observed comigration of progenitors of both lineages together through the fetal gut, and that each lineage contributed approximately one-half of the neurons of the postnatal hindgut ENS. Importantly, at least some terminal fates of the 2 lineages were distinct: CGRP^+^ mechanosensory neurons were overwhelmingly (>90% if not even 100%) derived from the placodes, whereas NOS^+^ inhibitory motor neurons were derived only from neural crest. Both lineages contributed to other types of NOS^+^ neurons and to other neuronal fates not characterized by CGRP or NOS expression. Lineage-specific mutation of the HSCR-associated gene *Ednrb* using either Cre driver caused HSCR, although for distinct cellular and mechanistic reasons.

The concept that the ENS is completely derived from neural crest is deeply entrenched. Although we presented abundant evidence that Pax2Cre is not active in early migrating neural crest and does not become active in even a subset of neural crest-derived ENS progenitors, a concern is that the conclusion of a placodal source of ENS progenitors rests on the recombination domain of a single driver line (Pax2Cre).

The *mSix1-21-NLSCre* transgene,^5^^,^^6^ abbreviated here as *Six1Cre*, couples an enhancer of the *Six1* gene to a minimal promoter. As previously documented, this enhancer restricts transgene expression to the embryonic placodes and their derived cranial nerves and to a small number of additional cranial tissues. The Six1Cre lineage does not include migratory neural crest-derived cells identified by Sox10 expression,^5^ which is true also for Pax2Cre.^3^

Here, we visualized the progeny of the Six1Cre lineage in the ENS. ENS progenitors labeled with Wnt1Cre (neural crest) or Pax2Cre (placode) both arise at embryonic day (E)9.5 at the postotic (vagal/nodose) level of the embryo.^3^ Six1Cre activity is mostly restricted to this same territory (Figure 1*A*). Six1Cre labels a subset of ectodermal cells in the second and third pharyngeal clefts, more limited than does Pax2Cre (Figure 1*B, C–H*). Six1Cre is not active in more anterior ectoderm nor in some mesodermal tissues in which Pax2Cre is active; the only overlap between the recombination domains of these 2 Cre lines is in the postotic placodes. Six1Cre, like Pax2Cre, is not active in neural crest-derived dorsal root ganglia along the entire body axis (Figure 1*A, B*), including the sacral domain (a possible source of ENS neurons).

**Figure 1:**
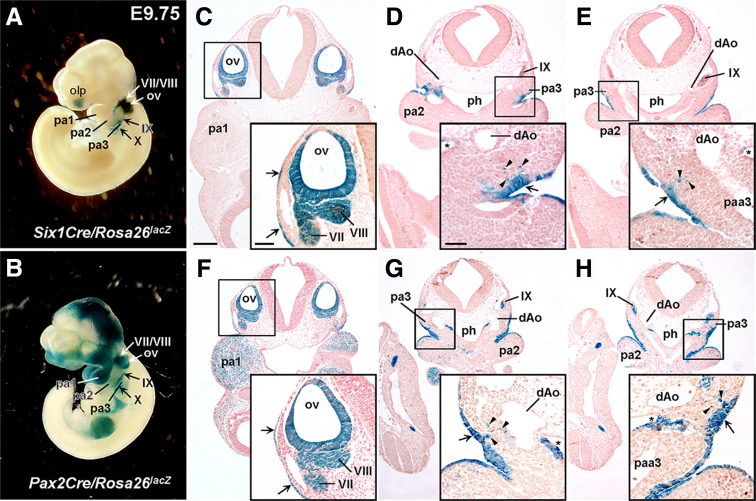
**Six1Cre and Pax2Cre label the early migratory ectodermal placode cell population.** (*A–B*) Wholemount X-gal staining of *Six1Cre/Rosa26*^*lacZ*^ (*A*) and *Pax2Cre/Rosa26*^*lacZ*^ (*B*) embryos at E9.75. (*C–E, F–H*) Transverse sections through the otic and epibranchial placodes of *Six1Cre/Rosa26*^*lacZ*^ (*C–E*) and *Pax2Cre/Rosa26*^*lacZ*^ (*F–H*) embryos at E9.75. Insets show magnified views of bracketed areas. Both Six1Cre and Pax2Cre label otic vesicle (otic placode-derived; *C, F*), epibranchial placode-derived CN VII/VIII (*C, F*) and CN IX (*D–E, G–H*) neurons, and the second (*C, F*) and third (*D–E, G–H*) pharyngeal arch ectoderm (also epibranchial placode-derived). *Arrows* denote lineage-labeled surface ectoderm (*C–E, F–H*); *arrowheads* point to lineage-labeled delaminating ectodermal placode cells (*D–E, G–H*); and *asterisks* denote Cre activity in subsets of pharyngeal pouch endoderm (*D–E, G–H*). dAo, dorsal aorta; olp, olfactory placode; ov, otic vesicle; pa, pharyngeal arch; paa, pharyngeal arch artery; ph, pharynx; VII, seventh (facial/geniculate) ganglion; VIII, eighth (vestibulocochlear) ganglion; IX, ninth (glossopharyngeal/petrosal) ganglion. Scale bars, 200 μm (*C–E, F–H*), 50 μm (insets *C, F*), 50 μm (insets *D–E, G–H*).

At E12.5, Six1Cre-labeled cells contributed to established placode fates in the inner ear and cranial nerves (Figure 2*A*; Supplementary Figure 1*A, B*). Six1Cre did not label any cells in neural crest-derived dorsal root and sympathetic ganglia or in smooth muscle cells of the pharyngeal arch arteries (Supplementary Figure 1*C*). Importantly, we detected Six1Cre-labeled cells among the p75^+^ enteric progenitors reaching the E12.5 hindgut (Figure 2*B*; Supplementary Figure 1*D*). In the newborn colon, lineage-labeled cells were a subset of ENS neurons expressing the pan-neuronal marker HuD (Figure 2*C*). At postnatal day (P)9, lineage-labeled cells were distributed across the distal colon (Figure 2*D*). We previously determined that Pax2Cre and Wnt1Cre each label approximately one-half of colonic ENS neurons in postnatal day P9 mice.^3^ Six1Cre only labeled 6% of postnatal colonic neurons (Figure 2*G* [*pie chart*]), per less extensive recombination in the postotic embryonic placodes (Figure 1). This subset of Six1Cre lineage-labeled ENS neurons were distributed approximately 20% to CGRP^+^ and NOS^+^ fates and 60% to other fates (Figure 2*E–F*; Figure 2*G* [*bar chart*]). In our previous study, Pax2Cre had essentially the same distribution of terminal fates, whereas fewer than 1% of Wnt1Cre-labeled neurons were CGRP^+^.^3^

**Figure 2:**
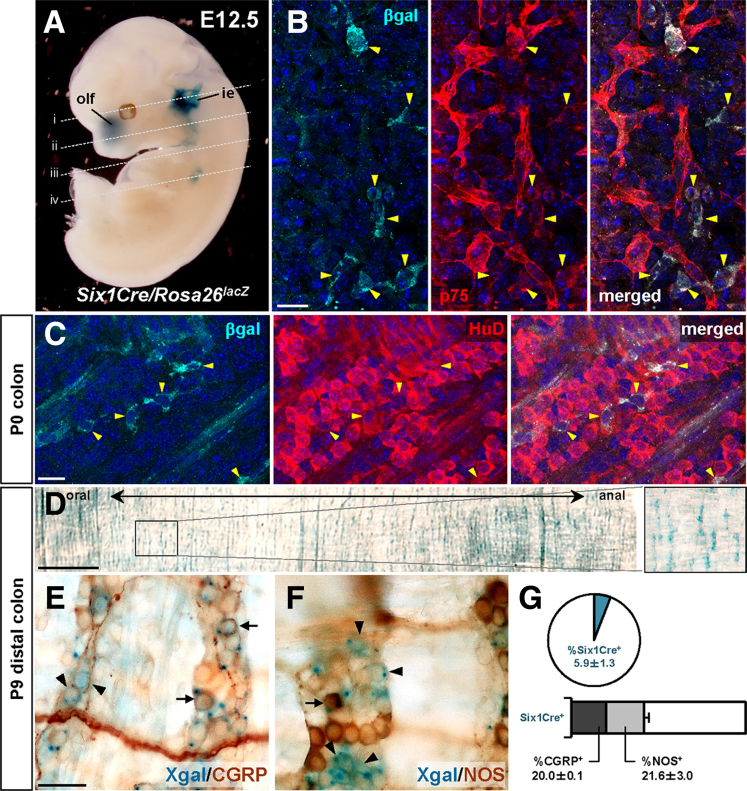
**Six1Cre-labeled epibranchial placode-derived cells contribute to the ENS.** (*A*) Wholemount X-gal staining of *Six1Cre/Rosa26*^*lacZ*^ at E12.5. Transverse sections corresponding to dotted lines (*i–iv*) are shown in Supplementary Figure 1*A–D*. (*B*) A confocal image of E12.5 gut isolated from *Six1Cre/Rosa26*^*lacZ*^ embryo stained for β-galactosidase (β-gal; *cyan*) and for ENS progenitor marker p75 (*red*). *Arrowheads* denote Six1Cre-labeled p75^+^ migratory ENS progenitor cells. (*C*) Wholemount preparations of P0 colon from *Six1Cre/Rosa26*^*lacZ*^ pup stained for bgal (*cyan*) and neuronal marker HuD (*red*). *Arrowheads* point to Six1Cre-derived neurons in the colonic myenteric plexus. (*D–F*) Wholemount preparations of distal colon isolated from P9 *Six1Cre/Rosa26*^*lacZ*^ mice stained with Xgal (*D–F*) and immunolabeled for CGRP (*E*) or NOS (*F*). A magnified view of the bracketed area in *D* is shown in inset (*right panel*). *Arrows* denote Six1Cre-labeled neurons co-expressing CGRP (*E*) or NOS (*F*) in myenteric ganglia. *Arrowheads* point to Six1Cre-labeled neurons lacking CGRP or NOS expression. (*G*) Compiled representation of Six1Cre lineage contribution to the colonic ENS. The pie chart represents the proportion of colonic ENS neurons labeled by Six1Cre. The bar chart shows the percentage of Six1Cre-labeled neurons that are positive for CGRP, NOS, or neither marker. These data represent 2282 myenteric neurons counted from distal colons isolated from 3 *Six1Cre/Rosa26*^*lacZ*^ mice (mean ± standard deviation). ie, inner ear; olf, olfactory epithelium. Scale bars: 20 μm (*B, C*), 1000 μm (*D*), 50 μm (*E–F*).

These results document Six1Cre as a second Cre line that recombines in the embryonic placodes and generates progeny that populate the mature ENS. These results augment the numerous lines of evidence based on Pax2Cre in normal and conditional mutant animals^3^ that the placodes are an authentic source of ENS progenitors distinct from the neural crest. A limitation of these studies is the absence of a non-Cre experimental approach to support this conclusion.

An important observation from our previous study was that the neural crest cell lineage had little or no plasticity to generate colonic CGRP^+^ mechanosensory neurons when these were absent in Pax2Cre conditional HSCR mutants. One candidate source of transplantable cells for HSCR is based on neural crest-directed differentiation of human induced pluripotent stem cells.^7^ Collectively, our results argue that cell-based therapeutic strategies for HSCR should ensure that transplanted cells are capable of yielding all cell types of the hindgut defecation circuitry.

## Supplementary Material

Supplementary Data

Extended
