# Supplementary material for: The Placode Lineage Contributes to the Enteric Nervous System: A Caution for Cell Transplantation Therapy for Hirschsprung Disease
Source: Cell Mol Gastroenterol Hepatol. 2025 Oct 3;20(2):101657. doi: 10.1016/j.jcmgh.2025.101657 (PMC12686883; doi:10.1016/j.jcmgh.2025.101657)
Supplement: Supplementary Data [file mmc1.pdf]

Supplemental Methods

Animals. *Six1Cre* [6] and *Rosa26*^{lacZ} [8]alleles have been described previously. All experiments with animals complied with the Institutional Regulation for Animal Experiment and Fundamental Guideline for Proper Conduct of Animal Experiment and Related Activities in Academic Research Institutions under the jurisdiction of the MEXT of Japan and approved by the Institutional Animal Experiment Committee of the Jichi Medical University.

Wholemount Xgal staining. Tissues isolated from *Six1Cre/Rosa26*^{lacZ} embryos and postnatal mice were fixed in 0.2% glutaraldehyde, 0.4% paraformaldehyde, 2mM $MgCl_2$ in PBS at 4°C overnight, then washed with 0.02% NP40, 0.01% sodium deoxycholate, 2mM $MgCl_2$ in PBS. X-gal staining was performed using 1mg/ml Xgal in 0.1M TrisHCl pH=7.4, 2mM $MgCl_2$, 0.02% NP40, 0.01% sodium deoxycholate, 5mM $K_3[Fe(CN)_6]$, 5mM $K_4[Fe(CN)_6]$ in PBS at room temperature overnight.

Wholemount immunofluorescence staining. Xgal-stained tissues were dehydrated in methanol sequence and bleached in 3% H_2O_2/20% DMSO/methanol solution overnight. After rehydration, tissues were cryoprotected in 30% sucrose/PBS, and permeablized by freeze-thaw cycles. Tissues were washed extensively with PBST (1% Tween-20 in PBS), and double immunofluorescence labelling was performed sequentially. First, tissues were incubated with primary antibody against β-galactosidase (chicken-polyclonal, 1:500, Abcam ab9361) in 2% BSA/PBST overnight at 37°C, and followed by Alexa-fluor conjugated secondary antibody

(Invitrogen) at 37°C for 4 hours. After extensive washes with PBST, tissues were incubated with primary antibody against p75 (goat polyclonal, 1:300, R&D AF1157) or HuD (moue monoclonal, 1:500, Santa Cruz sc-13577) in 1% non-fat dried milk, 20% DMSO in PBST for 2-3 days at 4°C, and followed by Alexa-fluor conjugated secondary antibody (Invitrogen) for 2-3 days at 4°C. Immunolabeled tissues were counterstained with DAPI and cleared with Sca*l*eU2 for confocal imaging.

Wholemount immunostaining. Glutaraldehyde-fixed colons (see wholemount Xgal staining method above) were first opened by longitudinal incision and the (inner) submucosal and mucosal layers removed prior to staining of the outer tissue (containing circular muscle, the myenteric plexus, longitudinal muscle, and serosa). Xgal-stained colonic preparations were permeabilized in PBST (1% Tween-20 in PBS), blocked in 1% non-fat dried milk, 20% DMSO in PBST, then incubated with primary antibody in blocking solution for 2-3 days at 4°C. Primary antibodies included rabbit anti-CGRP (1:500-1000, Millipore PC205L) and rabbit anti-NOS1 (1:500, Santa Cruz sc-648). After extensive washes with PBST, tissues were incubated with HRP-conjugated secondary antibody (1:200, Jackson Immunoresearch) for 2-3 days at 4°C. Immunoreactive signal was then visualized by DAB detection (0.2mg/ml DAB in PBST with 0.03% H2O2). Six1Cre$^+$, CGRP$^+$, NOS$^+$ and Six1Cre and CGRP (or NOS) double positive cells in the distal colon segments were counted using the Fiji ImageJ plugin Cell Counter.

Reference

8. Soriano P. Nat Genet 1999;21:70-1.

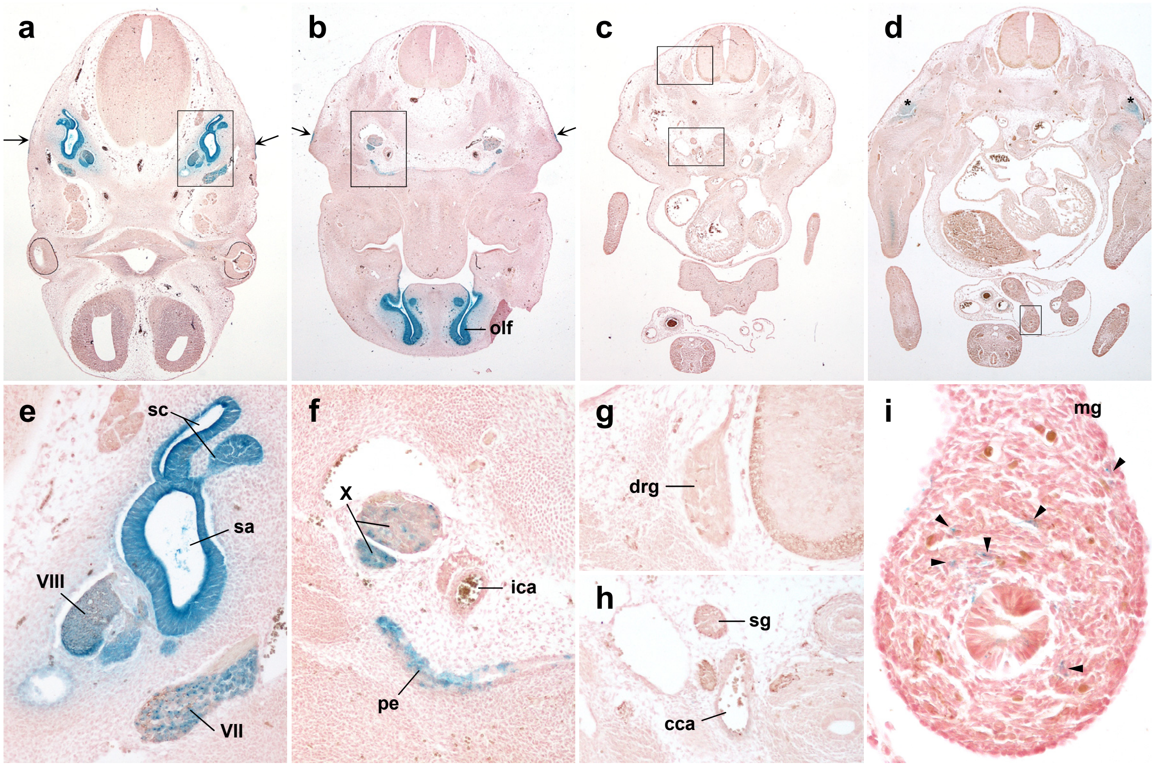

Supplemental Figure 1. Six1Cre activity during ENS progenitor migration and colonization. Transverse sections (a-d) corresponding to dotted lines (i-iv) of E12.5 *Six1Cre/Rosa26^{lacZ}* embryo shown in Figure 2a. Magnified views of bracketed area in a, b, c and d are shown in e, f, g-h, and i, respectively. **(a)** Cranial level (dotted line i): Six1Cre continues labeling the inner ear and subsets of facial (VII) and vestibulocochlear (VIII) nerves (see Fig. 1c). **(b)** Cervical level (dotted line ii): Six1Cre is active in subsets of vagal/nodose (X) ganglion. Lateral wall of pharyngeal endoderm is also Six1Cre^{+} (see Fig. 1d-e). **(c)** Thoracic level (dotted line iii): Six1Cre activity was not detected in any neural crest derived structures including cardiac outflow tract (cardiac neural crest-derived), and dorsal root (drg) and sympathetic ganglia (sg) (trunk neural crest-derived). **(d)** Limb level (dotted line iv): Six1Cre-labeled cells were detected in developing gut wall (arrowheads, i). Six1Cre also labeled subsets of limb muscle fibers (asterisks). Abbreviations: cca, common carotid artery; ica, internal carotid artery; sa, saccule; sc, semicircular canal. Scale bars, 200μm (c-e, f-h), 50μm (insets c, f), 50μm (insets d-e, g-h).
