# Supplementary material for: The Placode Lineage Contributes to the Enteric Nervous System: A Caution for Cell Transplantation Therapy for Hirschsprung Disease
Source: Cell Mol Gastroenterol Hepatol. 2025 Oct 3;20(2):101657. doi: 10.1016/j.jcmgh.2025.101657 (PMC12686883; doi:10.1016/j.jcmgh.2025.101657)
Supplement: Extended [file mmc2.pdf]

cmgh
CELLULAR AND MOLECULAR GASTROENTEROLOGY AND HEPATOLOGY

RESEARCH LETTERS

The Placode Lineage Contributes to the Enteric Nervous System: A Caution for Cell Transplantation Therapy for Hirschsprung Disease

The enteric nervous system (ENS) forms during development from progenitors that migrate through the fetal gut from cranial sites of origin. A deficiency in progenitor cell migration and gut colonization results in aganglionosis in the terminal bowel. After birth, when fecal progression through the hindgut requires a functional ENS circuitry, inadequate ENS function leads to inability to defecate and involuntary fecal retention. This condition is called Hirschsprung disease (HSCR). In the clinic, surgical resection of the aganglionic portion of the bowel is the only available treatment, although complications and persisting undesirable bowel function are common.[1]

Because HSCR is a developmental manifestation of incomplete neurogenic colonization, new strategies that involve transplantation of enteric progenitors into the aganglionic segment of the hindgut are under active investigation. For such cell-based therapies to successfully recreate a functional colonic circuitry, it is essential that the developmental biology of the hindgut ENS be properly understood. Cell transplantation approaches that do not incorporate the correct developmental insights may fail or may provide suboptimal outcomes.

The entirety of the ENS has long been thought to arise exclusively from the neural crest, a migratory population of multipotent progenitors that delaminate from the dorsal-most aspect of the neural tube during early development.[2] We recently provided evidence in mice that the embryonic placodes are a second and independent source of ENS progenitors.[3] Placodes are similar to neural crest in that both generate migratory neurons and other cell types,[4] but the placode lineage had never before been associated with the mammalian ENS. In our study,[3] we used Wnt1Cre to label the neural crest cell lineage, and Pax2Cre to label the placodal lineage. These Cre lines are very well-characterized and support highly efficient recombination that is authentic to the neural crest or placode lineages, as defined by many prior studies. We observed comigration of progenitors of both lineages together through the fetal gut, and that each lineage contributed approximately one-half of the neurons of the postnatal hindgut ENS. Importantly, at least some terminal fates of the 2 lineages were distinct: CGRP$^+$ mechanosensory neurons were overwhelmingly (>90% if not even 100%) derived from the placodes, whereas NOS$^+$ inhibitory motor neurons were derived only from neural crest. Both lineages contributed to other types of NOS$^+$ neurons and to other neuronal fates not characterized by CGRP or NOS expression. Lineage-specific mutation of the HSCR-associated gene *Ednrb* using either Cre driver caused HSCR, although for distinct cellular and mechanistic reasons.

The concept that the ENS is completely derived from neural crest is deeply entrenched. Although we presented abundant evidence that Pax2Cre is not active in early migrating neural crest and does not become active in even a subset of neural crest-derived ENS progenitors, a concern is that the conclusion of a placodal source of ENS progenitors rests on the recombination domain of a single driver line (Pax2Cre).

The *mSix1-21-NLSCre* transgene,[5,6] abbreviated here as *Six1Cre*, couples an enhancer of the *Six1* gene to a minimal promoter. As previously documented, this enhancer restricts transgene expression to the embryonic placodes and their derived cranial nerves and to a small number of additional cranial tissues. The Six1Cre lineage does not include migratory neural crest-derived cells identified by Sox10 expression,[5] which is true also for Pax2Cre.[3]

Here, we visualized the progeny of the Six1Cre lineage in the ENS. ENS progenitors labeled with Wnt1Cre (neural crest) or Pax2Cre (placode) both arise at embryonic day (E)9.5 at the postotic (vagal/nodose) level of the embryo.[3] Six1Cre activity is mostly restricted to this same territory (Figure 1*A*). Six1Cre labels a subset of ectodermal cells in the second and third pharyngeal clefts, more limited than does Pax2Cre (Figure 1*B, C–H*). Six1Cre is not active in more anterior ectoderm nor in some mesodermal tissues in which Pax2Cre is active; the only overlap between the recombination domains of these 2 Cre lines is in the postotic placodes. Six1Cre, like Pax2Cre, is not active in neural crest-derived dorsal root ganglia along the entire body axis (Figure 1*A, B*), including the sacral domain (a possible source of ENS neurons).

At E12.5, Six1Cre-labeled cells contributed to established placode fates in the inner ear and cranial nerves (Figure 2*A*; Supplementary Figure 1*A, B*). Six1Cre did not label any cells in neural crest-derived dorsal root and sympathetic ganglia or in smooth muscle cells of the pharyngeal arch arteries (Supplementary Figure 1*C*). Importantly, we detected Six1Cre-labeled cells among the p75$^+$ enteric progenitors reaching the E12.5 hindgut (Figure 2*B*; Supplementary Figure 1*D*). In the newborn colon, lineage-labeled cells were a subset of ENS neurons expressing the pan-neuronal marker HuD (Figure 2*C*). At postnatal day (P)9, lineage-labeled cells were distributed across the distal colon (Figure 2*D*). We previously determined that Pax2Cre and Wnt1Cre each label approximately one-half of colonic ENS neurons in postnatal day P9 mice.[3] Six1Cre only labeled 6% of postnatal colonic neurons (Figure 2*G* [*pie chart*]), per less extensive recombination in the postotic embryonic placodes (Figure 1). This subset of Six1Cre lineage-labeled ENS neurons were distributed approximately 20% to

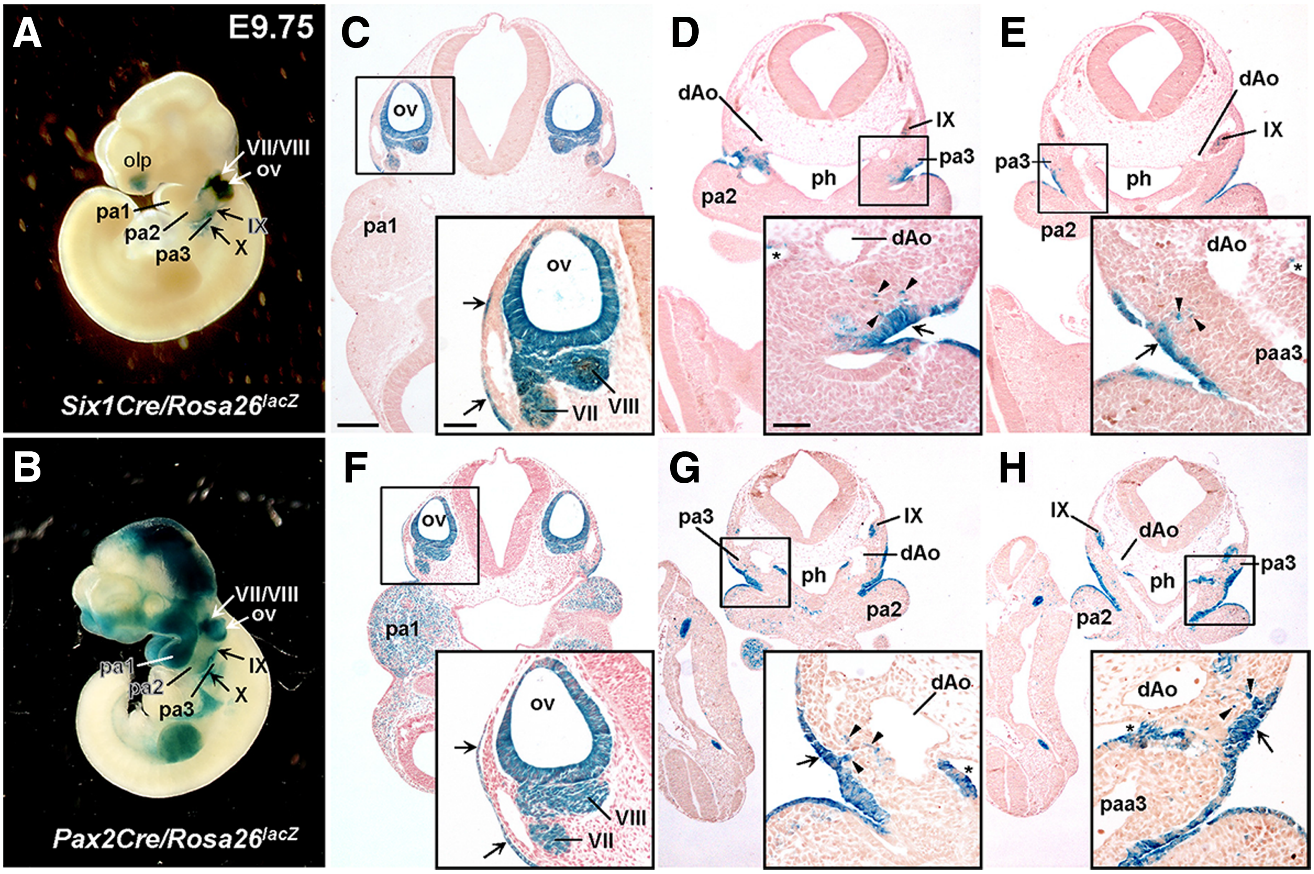

Figure 1. Six1Cre and Pax2Cre label the early migratory ectodermal placode cell population. (*A–B*) Wholemount X-gal staining of *Six1Cre/Rosa26^{lacZ}* (*A*) and *Pax2Cre/Rosa26^{lacZ}* (*B*) embryos at E9.75. (*C–E, F–H*) Transverse sections through the otic and epibranchial placodes of *Six1Cre/Rosa26^{lacZ}* (*C–E*) and *Pax2Cre/Rosa26^{lacZ}* (*F–H*) embryos at E9.75. Insets show magnified views of bracketed areas. Both Six1Cre and Pax2Cre label otic vesicle (otic placode-derived; *C, F*), epibranchial placode-derived CN VII/VIII (*C, F*) and CN IX (*D–E, G–H*) neurons, and the second (*C, F*) and third (*D–E, G–H*) pharyngeal arch ectoderm (also epibranchial placode-derived). *Arrows* denote lineage-labeled surface ectoderm (*C–E, F–H*); *arrowheads* point to lineage-labeled delaminating ectodermal placode cells (*D–E, G–H*); and *asterisks* denote Cre activity in subsets of pharyngeal pouch endoderm (*D–E, G–H*). dAo, dorsal aorta; olp, olfactory placode; ov, otic vesicle; pa, pharyngeal arch; paa, pharyngeal arch artery; ph, pharynx; VII, seventh (facial/geniculate) ganglion; VIII, eighth (vestibulocochlear) ganglion; IX, ninth (glossopharyngeal/petrosal) ganglion. Scale bars, 200 μm (*C–E, F–H*), 50 μm (insets *C, F*), 50 μm (insets *D–E, G–H*).

CGRP$^+$ and NOS$^+$ fates and 60% to other fates (Figure 2*E–F*; Figure 2*G* [*bar chart*]). In our previous study, Pax2Cre had essentially the same distribution of terminal fates, whereas fewer than 1% of Wnt1Cre-labeled neurons were CGRP$^+$.[3]

These results document Six1Cre as a second Cre line that recombines in the embryonic placodes and generates progeny that populate the mature ENS. These results augment the numerous lines of evidence based on Pax2Cre in normal and conditional mutant animals[3] that the placodes are an authentic source of ENS progenitors distinct from the neural crest. A limitation of these studies is the absence of a non-Cre experimental approach to support this conclusion.

An important observation from our previous study was that the neural crest cell lineage had little or no plasticity to generate colonic CGRP$^+$ mechanosensory neurons when these were absent in Pax2Cre conditional HSCR mutants. One candidate source of transplantable cells for HSCR is based on neural crest-directed differentiation of human induced pluripotent stem cells.[7] Collectively, our results argue that cell-based therapeutic strategies for HSCR should ensure that transplanted cells are capable of yielding all cell types of the hindgut defecation circuitry.

SHIGERU SATO
Division of Biology
Center for Molecular Medicine
Jichi Medical University
Shimotsuke, Tochigi, Japan

HENRY M. SUCOV
TAKAKO MAKITA
Department of Regenerative Medicine and Cell Biology
Medical University of South Carolina
Charleston, South Carolina

Supplementary Material

Note: To access the supplementary material accompanying this article, visit the full text version at https://doi.org/10.1016/j.jcmgh.2025.101657.

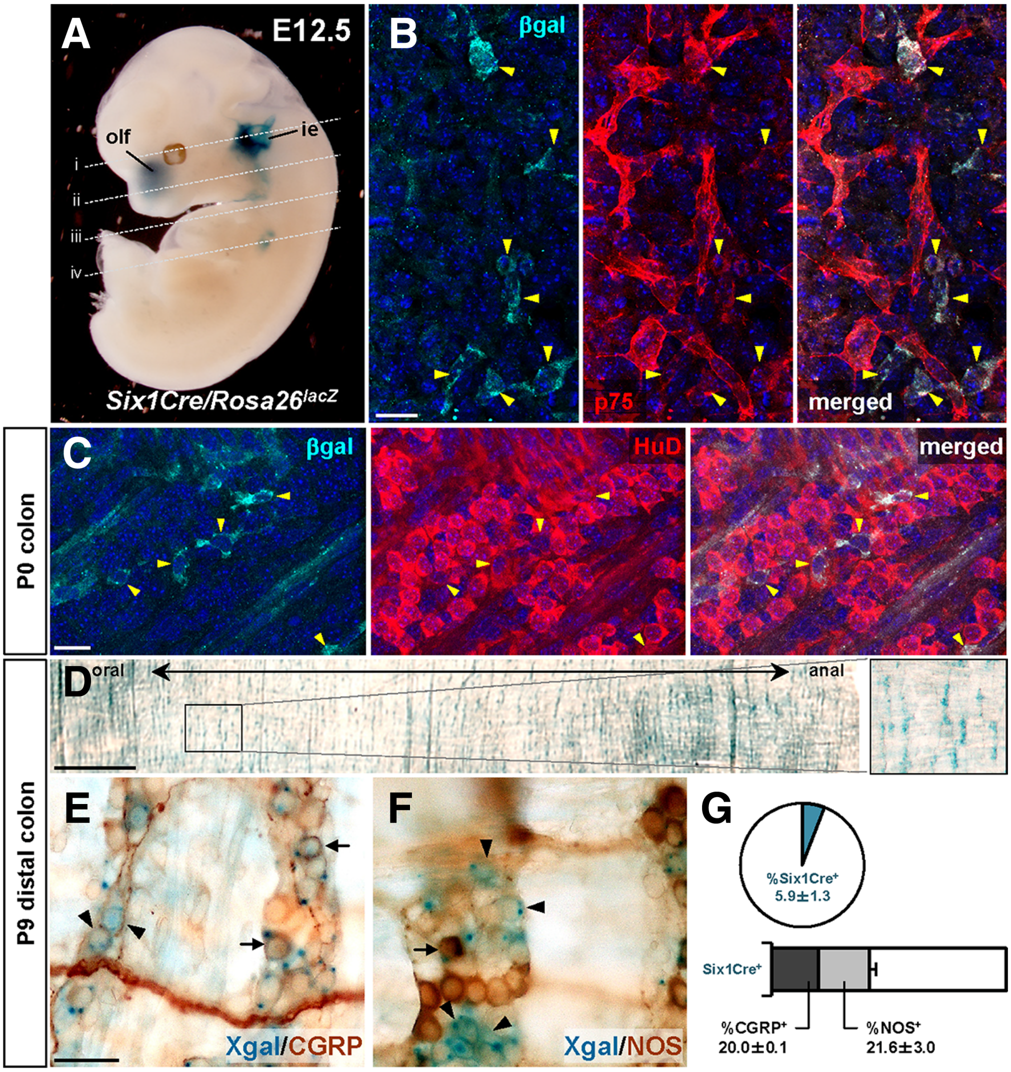

Figure 2. Six1Cre-labeled epibranchial placode-derived cells contribute to the ENS. (*A*) Wholemount X-gal staining of *Six1Cre/Rosa26*^{lacZ} at E12.5. Transverse sections corresponding to dotted lines (*i–iv*) are shown in Supplementary Figure 1*A–D*. (*B*) A confocal image of E12.5 gut isolated from *Six1Cre/Rosa26*^{lacZ} embryo stained for β-galactosidase (β-gal; *cyan*) and for ENS progenitor marker p75 (*red*). *Arrowheads* denote Six1Cre-labeled p75^{+} migratory ENS progenitor cells. (*C*) Wholemount preparations of P0 colon from *Six1Cre/Rosa26*^{lacZ} pup stained for bgal (*cyan*) and neuronal marker HuD (*red*). *Arrowheads* point to Six1Cre-derived neurons in the colonic myenteric plexus. (*D–F*) Wholemount preparations of distal colon isolated from P9 *Six1Cre/Rosa26*^{lacZ} mice stained with Xgal (*D–F*) and immunolabeled for CGRP (*E*) or NOS (*F*). A magnified view of the bracketed area in *D* is shown in inset (*right panel*). *Arrows* denote Six1Cre-labeled neurons co-expressing CGRP (*E*) or NOS (*F*) in myenteric ganglia. *Arrowheads* point to Six1Cre-labeled neurons lacking CGRP or NOS expression. (*G*) Compiled representation of Six1Cre lineage contribution to the colonic ENS. The pie chart represents the proportion of colonic ENS neurons labeled by Six1Cre. The bar chart shows the percentage of Six1Cre-labeled neurons that are positive for CGRP, NOS, or neither marker. These data represent 2282 myenteric neurons counted from distal colons isolated from 3 *Six1Cre/Rosa26*^{lacZ} mice (mean ± standard deviation). ie, inner ear; olf, olfactory epithelium. Scale bars: 20 μm (*B, C*), 1000 μm (*D*), 50 μm (*E–F*).

References

1. Burns AJ, et al. Dev Biol 2016; 417:229–251.
2. Le Douarin NM. The Neural Crest. Cambridge: Cambridge University Press, 1982.
3. Poltavski DM, et al. Elife 2024; 13:RP96424.
4. Baker CV, et al. Dev Biol 2001; 232:1–61.
5. Sato S, et al. Dev Dyn 2018; 247:250–261.
6. Sato S, et al. Dev Biol 2012; 368:95–108.
7. Barber K, et al. Nat Protoc 2019; 14:1261–1279.

Most current article

© 2025 The Authors. Published by Elsevier Inc. on behalf of the AGA Institute. This is an open access article under the CC BY license (http://creativecommons.org/licenses/by/4.0/).
2352-345X
https://doi.org/10.1016/j.jcmgh.2025.101657

Received December 26, 2024. Accepted September 26, 2025.

Correspondence
Address correspondence to: Takako Makita, PhD, Department of Regenerative Medicine and Cell Biology, Medical University of South Carolina, 173 Ashley Avenue, BSB630, MS #508, Charleston, South Carolina 29425. e-mail: makita@musc.edu; tel: (843) 792-2824.

Conflicts of interest
The authors disclose no conflicts.

Funding
This work was supported by grant NS084121 from the National Institutes of Health and in part by the Medical University of South Carolina Digestive Disease Research Core Center Pilot and Feasibility Program, National Institutes of Health P30 DK123704.

Data Availability
This study did not generate new unique reagents or original code. Raw data reported in this paper will be shared by the corresponding author upon request.

Cellular and Molecular Gastroenterology and Hepatology, Volume *20*

Supplemental information

The Placode Lineage Contributes to the Enteric Nervous System: A Caution for Cell Transplantation Therapy for Hirschsprung Disease

Shigeru Sato;Henry M. Sucov, and Takako Makita

Supplemental Methods

Animals. *Six1Cre* [6] and *Rosa26^{lacZ}* [8]alleles have been described previously. All experiments with animals complied with the Institutional Regulation for Animal Experiment and Fundamental Guideline for Proper Conduct of Animal Experiment and Related Activities in Academic Research Institutions under the jurisdiction of the MEXT of Japan and approved by the Institutional Animal Experiment Committee of the Jichi Medical University.

Wholemount Xgal staining. Tissues isolated from *Six1Cre/Rosa26^{lacZ}* embryos and postnatal mice were fixed in 0.2% glutaraldehyde, 0.4% paraformaldehyde, 2mM $MgCl_2$ in PBS at 4°C overnight, then washed with 0.02% NP40, 0.01% sodium deoxycholate, 2mM $MgCl_2$ in PBS. X-gal staining was performed using 1mg/ml Xgal in 0.1M TrisHCl pH=7.4, 2mM $MgCl_2$, 0.02% NP40, 0.01% sodium deoxycholate, 5mM $K_3[Fe(CN)_6]$, 5mM $K_4[Fe(CN)_6]$ in PBS at room temperature overnight.

Wholemount immunofluorescence staining. Xgal-stained tissues were dehydrated in methanol sequence and bleached in 3% H_2O_2/20% DMSO/methanol solution overnight. After rehydration, tissues were cryoprotected in 30% sucrose/PBS, and permeablized by freeze-thaw cycles. Tissues were washed extensively with PBST (1% Tween-20 in PBS), and double immunofluorescence labelling was performed sequentially. First, tissues were incubated with primary antibody against β-galactosidase (chicken-polyclonal, 1:500, Abcam ab9361) in 2% BSA/PBST overnight at 37°C, and followed by Alexa-fluor conjugated secondary antibody

(Invitrogen) at 37°C for 4 hours. After extensive washes with PBST, tissues were incubated with primary antibody against p75 (goat polyclonal, 1:300, R&D AF1157) or HuD (moue monoclonal, 1:500, Santa Cruz sc-13577) in 1% non-fat dried milk, 20% DMSO in PBST for 2-3 days at 4°C, and followed by Alexa-fluor conjugated secondary antibody (Invitrogen) for 2-3 days at 4°C. Immunolabeled tissues were counterstained with DAPI and cleared with Sca*l*eU2 for confocal imaging.

Wholemount immunostaining. Glutaraldehyde-fixed colons (see wholemount Xgal staining method above) were first opened by longitudinal incision and the (inner) submucosal and mucosal layers removed prior to staining of the outer tissue (containing circular muscle, the myenteric plexus, longitudinal muscle, and serosa). Xgal-stained colonic preparations were permeabilized in PBST (1% Tween-20 in PBS), blocked in 1% non-fat dried milk, 20% DMSO in PBST, then incubated with primary antibody in blocking solution for 2-3 days at 4°C. Primary antibodies included rabbit anti-CGRP (1:500-1000, Millipore PC205L) and rabbit anti-NOS1 (1:500, Santa Cruz sc-648). After extensive washes with PBST, tissues were incubated with HRP-conjugated secondary antibody (1:200, Jackson Immunoresearch) for 2-3 days at 4°C. Immunoreactive signal was then visualized by DAB detection (0.2mg/ml DAB in PBST with 0.03% H2O2). Six1Cre$^+$, CGRP$^+$, NOS$^+$ and Six1Cre and CGRP (or NOS) double positive cells in the distal colon segments were counted using the Fiji ImageJ plugin Cell Counter.

Reference

8. Soriano P. Nat Genet 1999;21:70-1.

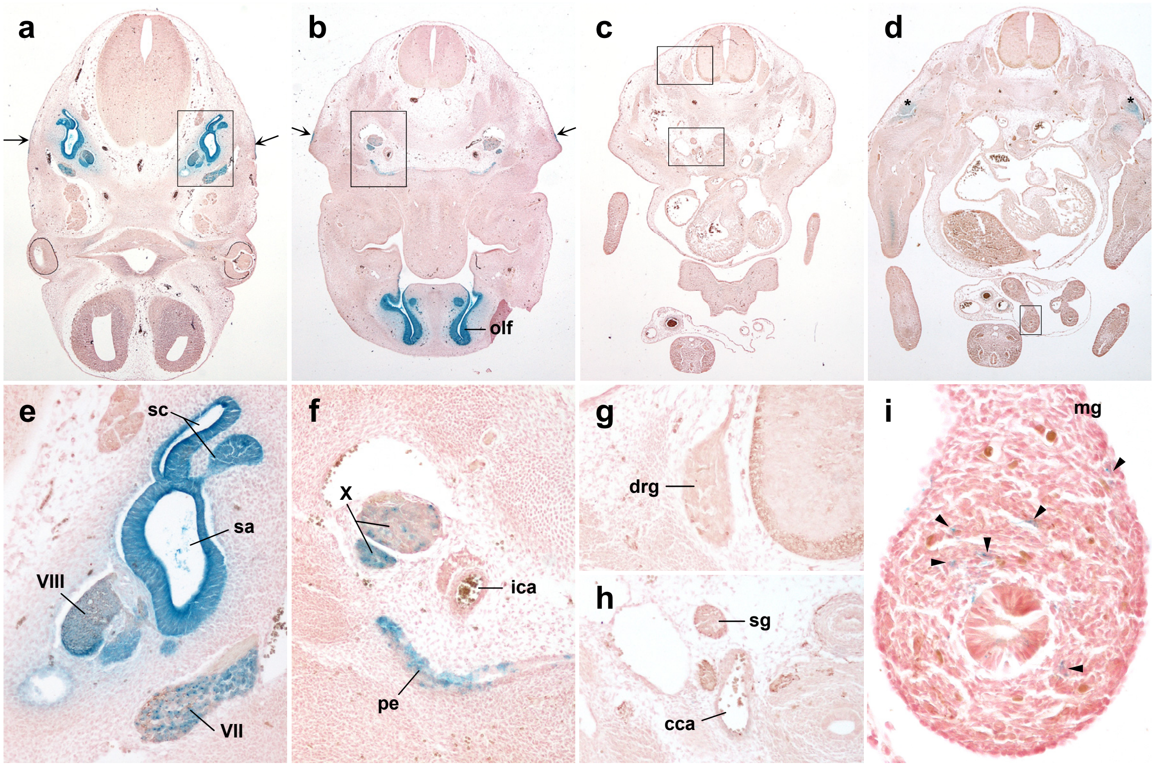

Supplemental Figure 1. Six1Cre activity during ENS progenitor migration and colonization. Transverse sections (a-d) corresponding to dotted lines (i-iv) of E12.5 *Six1Cre/Rosa26^{lacZ}* embryo shown in Figure 2a. Magnified views of bracketed area in a, b, c and d are shown in e, f, g-h, and i, respectively. **(a)** Cranial level (dotted line i): Six1Cre continues labeling the inner ear and subsets of facial (VII) and vestibulocochlear (VIII) nerves (see Fig. 1c). **(b)** Cervical level (dotted line ii): Six1Cre is active in subsets of vagal/nodose (X) ganglion. Lateral wall of pharyngeal endoderm is also Six1Cre^{+} (see Fig. 1d-e). **(c)** Thoracic level (dotted line iii): Six1Cre activity was not detected in any neural crest derived structures including cardiac outflow tract (cardiac neural crest-derived), and dorsal root (drg) and sympathetic ganglia (sg) (trunk neural crest-derived). **(d)** Limb level (dotted line iv): Six1Cre-labeled cells were detected in developing gut wall (arrowheads, i). Six1Cre also labeled subsets of limb muscle fibers (asterisks). Abbreviations: cca, common carotid artery; ica, internal carotid artery; sa, saccule; sc, semicircular canal. Scale bars, 200μm (c-e, f-h), 50μm (insets c, f), 50μm (insets d-e, g-h).

cmgh
CELLULAR AND MOLECULAR GASTROENTEROLOGY AND HEPATOLOGY

RESEARCH LETTERS

The Placode Lineage Contributes to the Enteric Nervous System: A Caution for Cell Transplantation Therapy for Hirschsprung Disease

The enteric nervous system (ENS) forms during development from progenitors that migrate through the fetal gut from cranial sites of origin. A deficiency in progenitor cell migration and gut colonization results in aganglionosis in the terminal bowel. After birth, when fecal progression through the hindgut requires a functional ENS circuitry, inadequate ENS function leads to inability to defecate and involuntary fecal retention. This condition is called Hirschsprung disease (HSCR). In the clinic, surgical resection of the aganglionic portion of the bowel is the only available treatment, although complications and persisting undesirable bowel function are common.[1]

Because HSCR is a developmental manifestation of incomplete neurogenic colonization, new strategies that involve transplantation of enteric progenitors into the aganglionic segment of the hindgut are under active investigation. For such cell-based therapies to successfully recreate a functional colonic circuitry, it is essential that the developmental biology of the hindgut ENS be properly understood. Cell transplantation approaches that do not incorporate the correct developmental insights may fail or may provide suboptimal outcomes.

The entirety of the ENS has long been thought to arise exclusively from the neural crest, a migratory population of multipotent progenitors that delaminate from the dorsal-most aspect of the neural tube during early development.[2] We recently provided evidence in mice that the embryonic placodes are a second and independent source of ENS progenitors.[3] Placodes are similar to neural crest in that both generate migratory neurons and other cell types,[4] but the placode lineage had never before been associated with the mammalian ENS. In our study,[3] we used Wnt1Cre to label the neural crest cell lineage, and Pax2Cre to label the placodal lineage. These Cre lines are very well-characterized and support highly efficient recombination that is authentic to the neural crest or placode lineages, as defined by many prior studies. We observed comigration of progenitors of both lineages together through the fetal gut, and that each lineage contributed approximately one-half of the neurons of the postnatal hindgut ENS. Importantly, at least some terminal fates of the 2 lineages were distinct: CGRP^{+} mechanosensory neurons were overwhelmingly (>90% if not even 100%) derived from the placodes, whereas NOS^{+} inhibitory motor neurons were derived only from neural crest. Both lineages contributed to other types of NOS^{+} neurons and to other neuronal fates not characterized by CGRP or NOS expression. Lineage-specific mutation of the HSCR-associated gene *Ednrb* using either Cre driver caused HSCR, although for distinct cellular and mechanistic reasons.

The concept that the ENS is completely derived from neural crest is deeply entrenched. Although we presented abundant evidence that Pax2Cre is not active in early migrating neural crest and does not become active in even a subset of neural crest-derived ENS progenitors, a concern is that the conclusion of a placodal source of ENS progenitors rests on the recombination domain of a single driver line (Pax2Cre).

The *mSix1-21-NLSCre* transgene,[5,6] abbreviated here as *Six1Cre*, couples an enhancer of the *Six1* gene to a minimal promoter. As previously documented, this enhancer restricts transgene expression to the embryonic placodes and their derived cranial nerves and to a small number of additional cranial tissues. The Six1Cre lineage does not include migratory neural crest-derived cells identified by Sox10 expression,[5] which is true also for Pax2Cre.[3]

Here, we visualized the progeny of the Six1Cre lineage in the ENS. ENS progenitors labeled with Wnt1Cre (neural crest) or Pax2Cre (placode) both arise at embryonic day (E)9.5 at the postotic (vagal/nodose) level of the embryo.[3] Six1Cre activity is mostly restricted to this same territory (Figure 1*A*). Six1Cre labels a subset of ectodermal cells in the second and third pharyngeal clefts, more limited than does Pax2Cre (Figure 1*B, C–H*). Six1Cre is not active in more anterior ectoderm nor in some mesodermal tissues in which Pax2Cre is active; the only overlap between the recombination domains of these 2 Cre lines is in the postotic placodes. Six1Cre, like Pax2Cre, is not active in neural crest-derived dorsal root ganglia along the entire body axis (Figure 1*A, B*), including the sacral domain (a possible source of ENS neurons).

At E12.5, Six1Cre-labeled cells contributed to established placode fates in the inner ear and cranial nerves (Figure 2*A*; Supplementary Figure 1*A, B*). Six1Cre did not label any cells in neural crest-derived dorsal root and sympathetic ganglia or in smooth muscle cells of the pharyngeal arch arteries (Supplementary Figure 1*C*). Importantly, we detected Six1Cre-labeled cells among the p75^{+} enteric progenitors reaching the E12.5 hindgut (Figure 2*B*; Supplementary Figure 1*D*). In the newborn colon, lineage-labeled cells were a subset of ENS neurons expressing the pan-neuronal marker HuD (Figure 2*C*). At postnatal day (P)9, lineage-labeled cells were distributed across the distal colon (Figure 2*D*). We previously determined that Pax2Cre and Wnt1Cre each label approximately one-half of colonic ENS neurons in postnatal day P9 mice.[3] Six1Cre only labeled 6% of postnatal colonic neurons (Figure 2*G* [*pie chart*]), per less extensive recombination in the

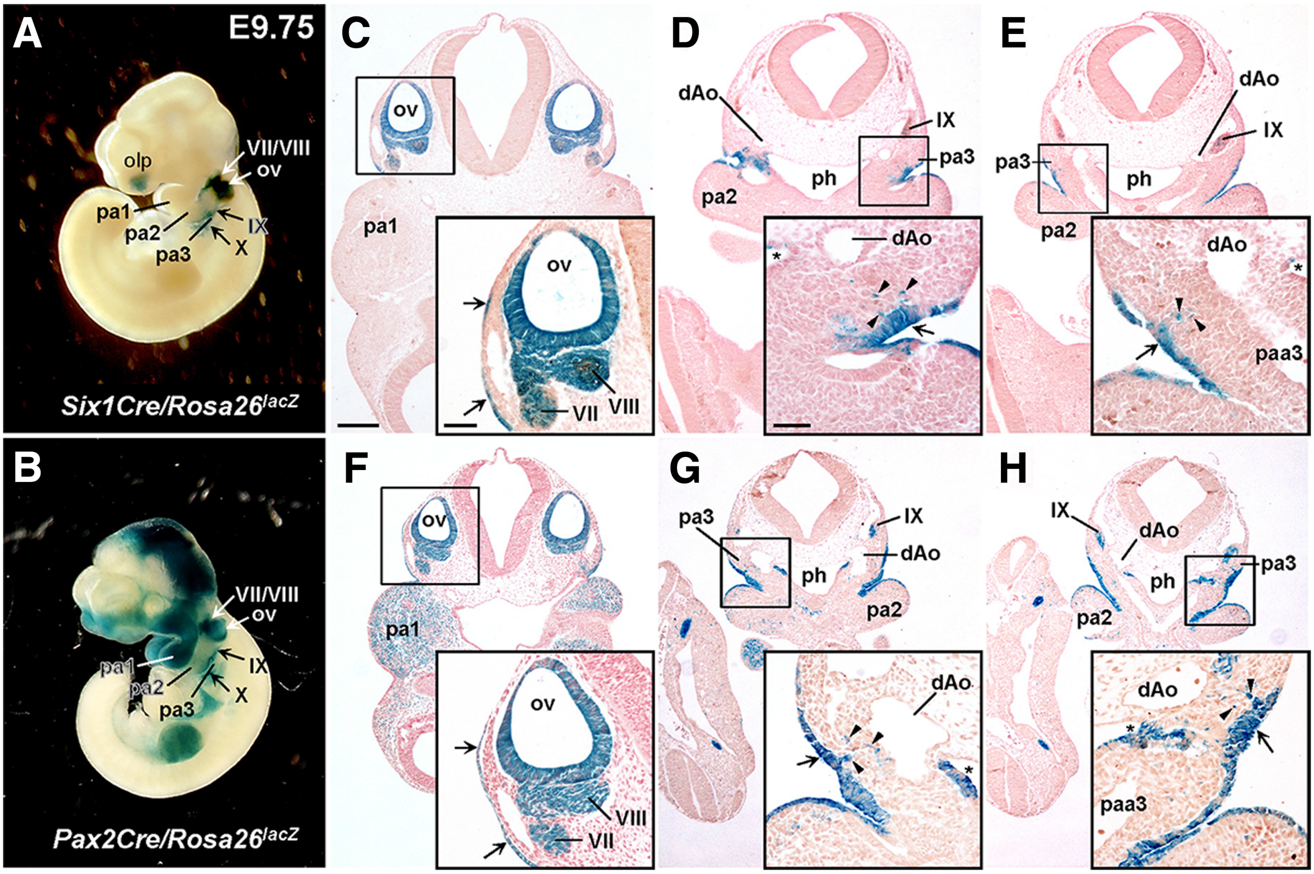

Figure 1. Six1Cre and Pax2Cre label the early migratory ectodermal placode cell population. (*A–B*) Wholemount X-gal staining of *Six1Cre/Rosa26*^{lacZ} (*A*) and *Pax2Cre/Rosa26*^{lacZ} (*B*) embryos at E9.75. (*C–E, F–H*) Transverse sections through the otic and epibranchial placodes of *Six1Cre/Rosa26*^{lacZ} (*C–E*) and *Pax2Cre/Rosa26*^{lacZ} (*F–H*) embryos at E9.75. Insets show magnified views of bracketed areas. Both Six1Cre and Pax2Cre label otic vesicle (otic placode-derived; *C, F*), epibranchial placode-derived CN VII/VIII (*C, F*) and CN IX (*D–E, G–H*) neurons, and the second (*C, F*) and third (*D–E, G–H*) pharyngeal arch ectoderm (also epibranchial placode-derived). *Arrows* denote lineage-labeled surface ectoderm (*C–E, F–H*); *arrowheads* point to lineage-labeled delaminating ectodermal placode cells (*D–E, G–H*); and *asterisks* denote Cre activity in subsets of pharyngeal pouch endoderm (*D–E, G–H*). dAo, dorsal aorta; olp, olfactory placode; ov, otic vesicle; pa, pharyngeal arch; paa, pharyngeal arch artery; ph, pharynx; VII, seventh (facial/geniculate) ganglion; VIII, eighth (vestibulocochlear) ganglion; IX, ninth (glossopharyngeal/petrosal) ganglion. Scale bars, 200 μm (*C–E, F–H*), 50 μm (insets *C, F*), 50 μm (insets *D–E, G–H*).

postotic embryonic placodes (Figure 1). This subset of Six1Cre lineage-labeled ENS neurons were distributed approximately 20% to CGRP$^+$ and NOS$^+$ fates and 60% to other fates (Figure 2*E–F*; Figure 2*G* [*bar chart*]). In our previous study, Pax2Cre had essentially the same distribution of terminal fates, whereas fewer than 1% of Wnt1Cre-labeled neurons were CGRP$^+$.[3]

These results document Six1Cre as a second Cre line that recombines in the embryonic placodes and generates progeny that populate the mature ENS. These results augment the numerous lines of evidence based on Pax2Cre in normal and conditional mutant animals[3] that the placodes are an authentic source of ENS progenitors distinct from the neural crest. A limitation of these studies is the absence of a non-Cre experimental approach to support this conclusion.

An important observation from our previous study was that the neural crest cell lineage had little or no plasticity to generate colonic CGRP$^+$ mechanosensory neurons when these were absent in Pax2Cre conditional HSCR mutants. One candidate source of transplantable cells for HSCR is based on neural crest-directed differentiation of human induced pluripotent stem cells.[7] Collectively, our results argue that cell-based therapeutic strategies for HSCR should ensure that transplanted cells are capable of yielding all cell types of the hindgut defecation circuitry.

SHIGERU SATO
Division of Biology
Center for Molecular Medicine
Jichi Medical University
Shimotsuke, Tochigi, Japan

HENRY M. SUCOV
TAKAKO MAKITA
Department of Regenerative Medicine and Cell Biology
Medical University of South Carolina
Charleston, South Carolina

Supplementary Material

Note: To access the supplementary material accompanying this article, visit

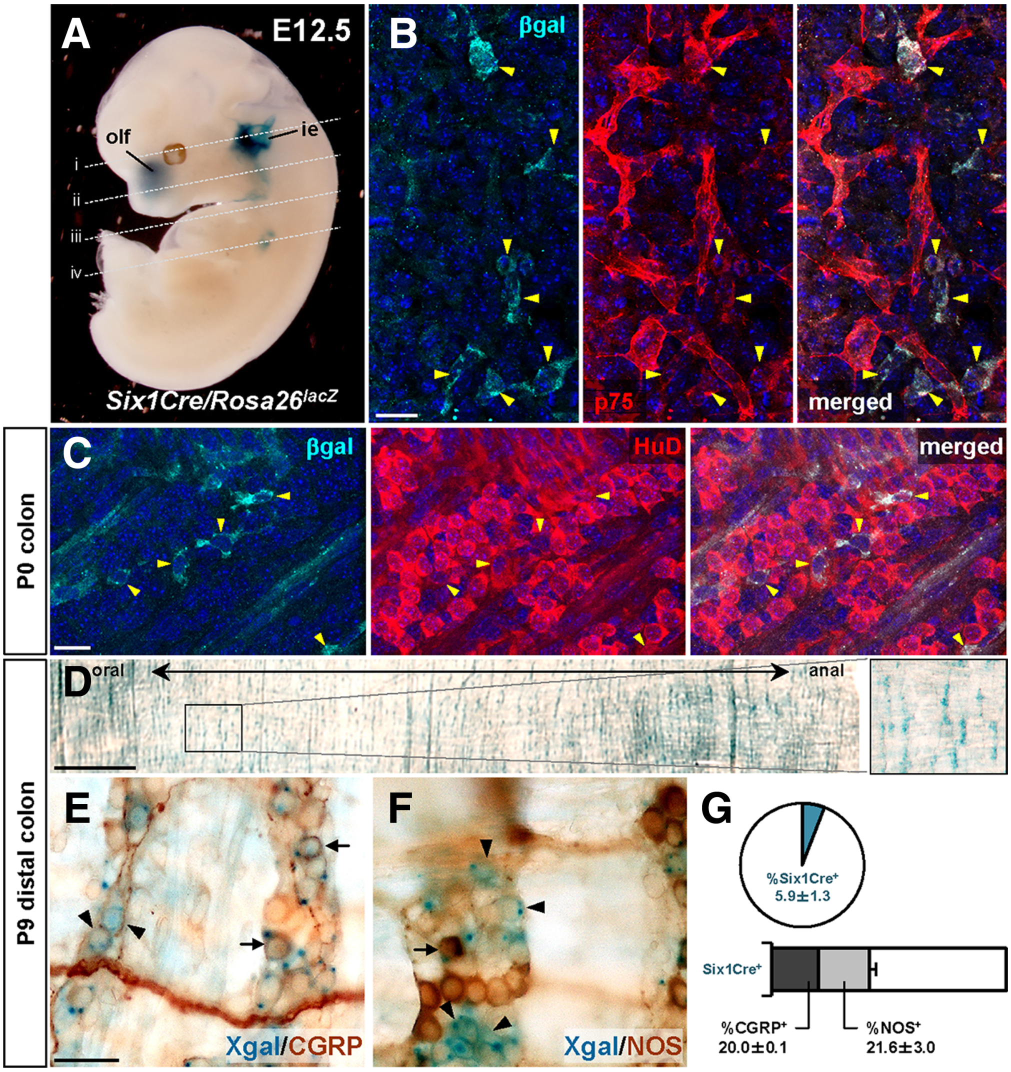

Figure 2. Six1Cre-labeled epibranchial placode-derived cells contribute to the ENS. (*A*) Wholemount X-gal staining of *Six1Cre/Rosa26*^{lacZ} at E12.5. Transverse sections corresponding to dotted lines (*i–iv*) are shown in Supplementary Figure 1*A–D*. (*B*) A confocal image of E12.5 gut isolated from *Six1Cre/Rosa26*^{lacZ} embryo stained for β-galactosidase (β-gal; *cyan*) and for ENS progenitor marker p75 (*red*). *Arrowheads* denote Six1Cre-labeled p75^{+} migratory ENS progenitor cells. (*C*) Wholemount preparations of P0 colon from *Six1Cre/Rosa26*^{lacZ} pup stained for bgal (*cyan*) and neuronal marker HuD (*red*). *Arrowheads* point to Six1Cre-derived neurons in the colonic myenteric plexus. (*D–F*) Wholemount preparations of distal colon isolated from P9 *Six1Cre/Rosa26*^{lacZ} mice stained with Xgal (*D–F*) and immunolabeled for CGRP (*E*) or NOS (*F*). A magnified view of the bracketed area in *D* is shown in inset (*right panel*). *Arrows* denote Six1Cre-labeled neurons co-expressing CGRP (*E*) or NOS (*F*) in myenteric ganglia. *Arrowheads* point to Six1Cre-labeled neurons lacking CGRP or NOS expression. (*G*) Compiled representation of Six1Cre lineage contribution to the colonic ENS. The pie chart represents the proportion of colonic ENS neurons labeled by Six1Cre. The bar chart shows the percentage of Six1Cre-labeled neurons that are positive for CGRP, NOS, or neither marker. These data represent 2282 myenteric neurons counted from distal colons isolated from 3 *Six1Cre/Rosa26*^{lacZ} mice (mean ± standard deviation). ie, inner ear; olf, olfactory epithelium. Scale bars: 20 μm (*B, C*), 1000 μm (*D*), 50 μm (*E–F*).

the full text version at https://doi.org/10.1016/j.jcmgh.2025.101657.

References

1. Burns AJ, et al. Dev Biol 2016;417:229–251.
2. Le Douarin NM. The Neural Crest. Cambridge: Cambridge University Press, 1982.
3. Poltavski DM, et al. Elife 2024;13:RP96424.
4. Baker CV, et al. Dev Biol 2001;232:1–61.
5. Sato S, et al. Dev Dyn 2018;247:250–261.
6. Sato S, et al. Dev Biol 2012;368:95–108.
7. Barber K, et al. Nat Protoc 2019;14:1261–1279.

Most current article

© 2025 The Authors. Published by Elsevier Inc. on behalf of the AGA Institute. This is an open access article under the CC BY license (http://creativecommons.org/licenses/by/4.0/).
2352-345X
https://doi.org/10.1016/j.jcmgh.2025.101657

Received December 26, 2024. Accepted September 26, 2025.

Correspondence
Address correspondence to: Takako Makita, PhD, Department of Regenerative Medicine and Cell Biology, Medical University of South Carolina, 173 Ashley Avenue, BSB630, MS #508, Charleston, South Carolina 29425. e-mail: makita@musc.edu; tel: (843) 792-2824.

Conflicts of interest
The authors disclose no conflicts.

Funding
This work was supported by grant NS084121 from the National Institutes of Health and in part by the Medical University of South Carolina Digestive Disease Research Core Center Pilot and Feasibility Program, National Institutes of Health P30 DK123704.

Data Availability
This study did not generate new unique reagents or original code. Raw data reported in this paper will be shared by the corresponding author upon request.

Cellular and Molecular Gastroenterology and Hepatology, Volume *20*

Supplemental information

The Placode Lineage Contributes to the Enteric Nervous System: A Caution for Cell Transplantation Therapy for Hirschsprung Disease

Shigeru Sato;Henry M. Sucov, and Takako Makita

Supplemental Methods

Animals. *Six1Cre*[6] and *Rosa26*^{lacZ}[8]alleles have been described previously. All experiments with animals complied with the Institutional Regulation for Animal Experiment and Fundamental Guideline for Proper Conduct of Animal Experiment and Related Activities in Academic Research Institutions under the jurisdiction of the MEXT of Japan and approved by the Institutional Animal Experiment Committee of the Jichi Medical University.

Wholemount Xgal staining. Tissues isolated from *Six1Cre/Rosa26*^{lacZ} embryos and postnatal mice were fixed in 0.2% glutaraldehyde, 0.4% paraformaldehyde, 2mM $MgCl_2$ in PBS at 4°C overnight, then washed with 0.02% NP40, 0.01% sodium deoxycholate, 2mM $MgCl_2$ in PBS. X-gal staining was performed using 1mg/ml Xgal in 0.1M TrisHCl pH=7.4, 2mM $MgCl_2$, 0.02% NP40, 0.01% sodium deoxycholate, 5mM $K_3[Fe(CN)_6]$, 5mM $K_4[Fe(CN)_6]$ in PBS at room temperature overnight.

Wholemount immunofluorescence staining. Xgal-stained tissues were dehydrated in methanol sequence and bleached in 3% H_2O_2/20% DMSO/methanol solution overnight. After rehydration, tissues were cryoprotected in 30% sucrose/PBS, and permeablized by freeze-thaw cycles. Tissues were washed extensively with PBST (1% Tween-20 in PBS), and double immunofluorescence labelling was performed sequentially. First, tissues were incubated with primary antibody against β-galactosidase (chicken-polyclonal, 1:500, Abcam ab9361) in 2% BSA/PBST overnight at 37°C, and followed by Alexa-fluor conjugated secondary antibody

(Invitrogen) at 37°C for 4 hours. After extensive washes with PBST, tissues were incubated with primary antibody against p75 (goat polyclonal, 1:300, R&D AF1157) or HuD (moue monoclonal, 1:500, Santa Cruz sc-13577) in 1% non-fat dried milk, 20% DMSO in PBST for 2-3 days at 4°C, and followed by Alexa-fluor conjugated secondary antibody (Invitrogen) for 2-3 days at 4°C. Immunolabeled tissues were counterstained with DAPI and cleared with Sca*l*eU2 for confocal imaging.

Wholemount immunostaining. Glutaraldehyde-fixed colons (see wholemount Xgal staining method above) were first opened by longitudinal incision and the (inner) submucosal and mucosal layers removed prior to staining of the outer tissue (containing circular muscle, the myenteric plexus, longitudinal muscle, and serosa). Xgal-stained colonic preparations were permeabilized in PBST (1% Tween-20 in PBS), blocked in 1% non-fat dried milk, 20% DMSO in PBST, then incubated with primary antibody in blocking solution for 2-3 days at 4°C. Primary antibodies included rabbit anti-CGRP (1:500-1000, Millipore PC205L) and rabbit anti-NOS1 (1:500, Santa Cruz sc-648). After extensive washes with PBST, tissues were incubated with HRP-conjugated secondary antibody (1:200, Jackson Immunoresearch) for 2-3 days at 4°C. Immunoreactive signal was then visualized by DAB detection (0.2mg/ml DAB in PBST with 0.03% H2O2). Six1Cre$^+$, CGRP$^+$, NOS$^+$ and Six1Cre and CGRP (or NOS) double positive cells in the distal colon segments were counted using the Fiji ImageJ plugin Cell Counter.

Reference

8. Soriano P. Nat Genet 1999;21:70-1.

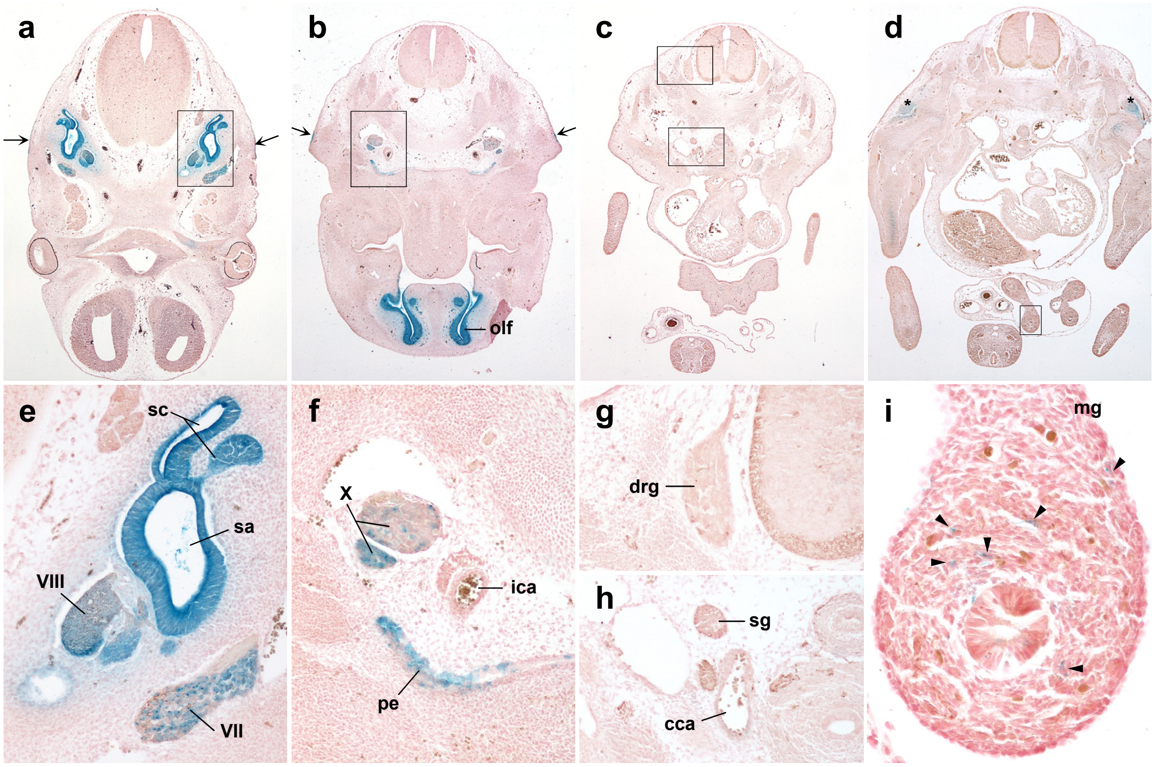

Supplemental Figure 1. Six1Cre activity during ENS progenitor migration and colonization. Transverse sections (a-d) corresponding to dotted lines (i-iv) of E12.5 *Six1Cre/Rosa26^{lacZ}* embryo shown in Figure 2a. Magnified views of bracketed area in a, b, c and d are shown in e, f, g-h, and i, respectively. **(a)** Cranial level (dotted line i): Six1Cre continues labeling the inner ear and subsets of facial (VII) and vestibulocochlear (VIII) nerves (see Fig. 1c). **(b)** Cervical level (dotted line ii): Six1Cre is active in subsets of vagal/nodose (X) ganglion. Lateral wall of pharyngeal endoderm is also Six1Cre^{+} (see Fig. 1d-e). **(c)** Thoracic level (dotted line iii): Six1Cre activity was not detected in any neural crest derived structures including cardiac outflow tract (cardiac neural crest-derived), and dorsal root (drg) and sympathetic ganglia (sg) (trunk neural crest-derived). **(d)** Limb level (dotted line iv): Six1Cre-labeled cells were detected in developing gut wall (arrowheads, i). Six1Cre also labeled subsets of limb muscle fibers (asterisks). Abbreviations: cca, common carotid artery; ica, internal carotid artery; sa, saccule; sc, semicircular canal. Scale bars, 200μm (c-e, f-h), 50μm (insets c, f), 50μm (insets d-e, g-h).
